# Novel mutation in the *MED23* gene for intellectual disability: A case report and literature review

**DOI:** 10.1002/ccr3.1942

**Published:** 2019-01-09

**Authors:** Feyzollah Hashemi‐Gorji, Majid Fardaei, Seyed Mohammad Bagher Tabei, Mohammad Miryounesi

**Affiliations:** ^1^ Genomic Research Center Shahid Beheshti University of Medical Sciences Tehran Iran; ^2^ Department of Medical Genetics, School of Medicine Shiraz University of Medical Sciences Shiraz Iran

**Keywords:** intellectual disability, mental retardation, microcephaly

## Abstract

MED23 deficiency causes the autosomal recessive Intellectual Disability (ID). Here we report an Iranian case with nonsyndromic ID presenting with developmental delay, microcephaly, hypotonia, severe ID, speech delay, and spasticity, who was homozygous for the novel MED23 c.670C>G variant. These results expand the clinical and mutation spectrum of MED23 deficiency.

## 1 BACKGROUND

Intellectual disability (ID) is a clinical and genetic heterogeneous disorder, characterized by a significant limitation in intellectual functioning and adaptive behavior.^1^ The prevalence of ID is estimated to affect 1%‐3% of the general population.^2^, ^3^ So far, more than 450 genes have been identified to cause ID.^4^ Previous study has shown that the transcriptional dysregulation of Mediator genes have a crucial role in the brain development and its functioning.^5^ The *MED23 *(OMIM 605042) gene encodes a subunit of the Mediator complex, which integrates various signaling pathways.^6^, ^7^, ^8^ Affected patients manifest with axial hypotonia, choreoathetosis, dystonia, Global Developmental Delay (GDD), mental retardation, microcephaly, mild to severe ID, spasticity, with or without seizure. The MRI of the brain may be normal or it may show temporal lobe hypomyelination, delayed myelination, pontine hypoplasia, and thin corpus callosum.^5^, ^9^, ^10^ In this study, we develop a report on an Iranian case, diagnosed with severe nonsyndromic intellectual disabilities.

## 2 METHODS

### 2.1 Case presentation

**Figure 1:**
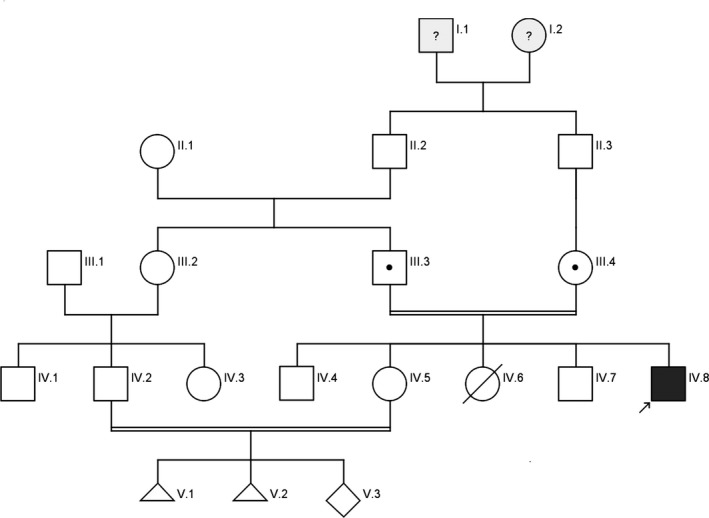
The pedigree of the index patient

The patient (IV.8, Figure 1) was a 25‐year‐old male, born healthy at birth to a first cousin parents. He was the fifth born child in the family and was normal up to one and half years of age. Initial symptoms of the disease, as noticed, were as developmental delay, microcephaly, difficulty in walking due to spasticity, and speech delay. The MRI of the brain and the electroencephalogram (EEG) were not performed on the patient. A clinical examination at the age of 24 revealed hypotonia in the patient's hands, injuries due to skin lesions, mental retardation, microcephaly, a minor problem in walking, and difficulty in speech. Molecular tests for the Fragile X syndrome and karyotype were normal. There was no family history of mental retardation. Due to the current pregnancy of the patient's sister (IV.5, Figure 1), who also was in a consanguineous marriage, the family requested genetic investigations, and we performed clinical Whole Exome Sequencing (WES).

### 2.2 Mutation analysis

This study was approved by the Ethical Committee, Deputy of Research Affairs of Shahid Beheshti University of Medical Sciences, which follows the tenets of the Declaration of Helsinki. Blood samples were collected from the patient and his family after written and informed consent was obtained. The SureSelectXT V5+UTRs Kit (Agilent Technologies, Lake forest, CA, USA) was used for the enrichment of genomic DNA. Sequencing was then performed with 101 bp paired end using the HiSeq2500 (Illumina, San Diego, CA, USA) Sequencer. The Readings were aligned to the human reference assembly (UCSC hg19, NCBI build 37.1) with the Burrows‐Wheeler Aligner (BWA v.0.5.8). The Picard tool (version 1.118) was used to remove PCR duplications. Single‐Nucleotide Polymorphism (SNP) and indel were detected with the Genome Analysis Toolkit (GATK). Furthermore, variants were annotated using the ANNOVAR software tool and were filtered based on quality, frequency <0.001 in the 1000 genome project, ESP6500, NHBL Exome Variant Server (EVS), ExAC, dbSNP 138, Iranome (www.iranome.com), and an in‐house Iranian exome dataset (500 individuals), protein effect, pathogenicity and previous associations with the phenotype. Sanger sequencing was used for confirmation of identified variant in the patient and parents, brothers, and one sister. Several prediction tools were used to predict the pathogenicity of the identified variant.^11^, ^12^, ^13^, ^14^, ^15^ Protein modeling was then performed to predict tertiary structure of wild type and mutant human MED23 using HOPE, Phyre 2, Swiss modeling, and RaptorX servers.^16^, ^17^, ^18^, ^19^, ^20^

## 3 RESULTS

**Figure 2:**
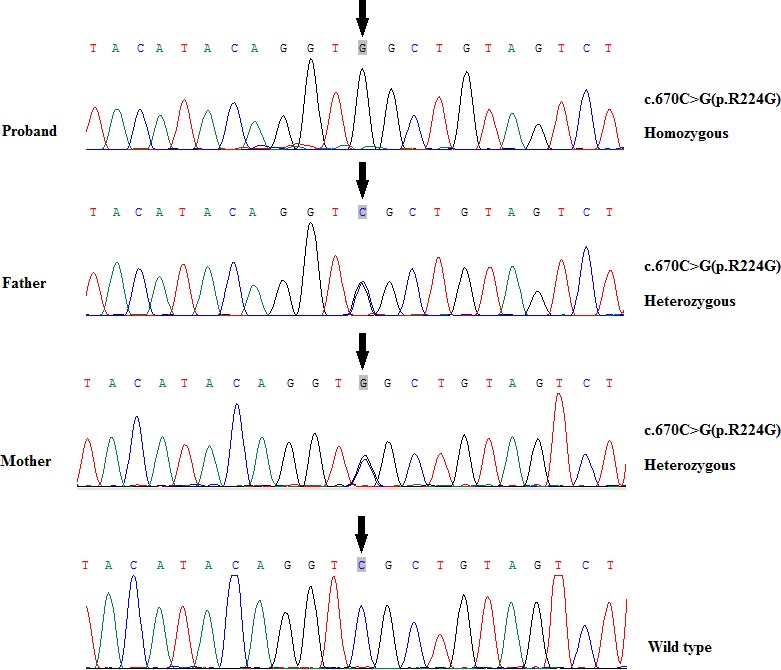
Genomic DNA analysis of the patient show a homozygous missense variant, C.670C>G (P.R224G), while both parents are unaffected heterozygous carriers, brothers and sister as wild‐type homozygous

The WES results ascertained one novel homozygous missense variant, NM_015979.3:c.670C>G (NP_057063.2:p.R224G) (Table 1), located in the *MED23* gene. This variant was neither found in the 1000 Genome project, ESP6500, EVS, ExAC, Iranome, and in‐house Iranian exome data. Sanger sequencing result confirmed the patient as homozygous (Figure 2), patient's parents as carrier, sister and brothers as wild‐type homozygous for the variant c.670C>G. The variant was predicted to be possibly damaging by PolyPhen2, damaging by SIFT, deleterious by PROVEN, pathogenic by UMD‐Predictor, and disease‐causing by the Mutation Taster. The in‐silico analysis result showed that the position 224 is a conserved amino acid and is substituted with polar amino acids, Arginine in 20 species and Histidine in 27 species. The variant c.670C>G is located in the early exonic position and was also predicted to potentially alter the splice site using Human Splice Finder 3 (HSF 3.0). Owing to the lack of structural information on the *MED23* gene, we were unable to develop the modeling for p.R224G variant on the protein. According to ACMG guideline this variant was classified as Variant of Uncertain significance (VUS).^21^

**Table 1:** Summary of identified variant in the *MED23* gene associated with neurological disorder

Variant	Codon	SNP	consanguinity	Clinical features	Zygosity	CADD score	Reference
c.670C>G	R224G		Yes	Developmental delay, hypotonia in hands, microcephaly, sever ID, speech delay, spasticity	Homozygous	26.4	This study
c.1850G>A	R617Q	rs745997916	Yes	Mild to moderate ID	Homozygous	35	Hashimoto et al^5^
c.1937A>G	Q646R	rs745997916	Yes	GDD, microcephaly, axial hypotonia, spasticity, refractory seizure	Homozygous	28	Lionel et al^10^
c.3656A>G	H1219R	rs527236035	No	Severe ID, axial hypotonia, choreoathetosis, dystonia spasticity	Heterozygous	23	Trehan et al^9^
c.4006C>T	R1336X	rs527236036			Heterozygous	51	

CADD, Combined Annotation Dependent Depletion; GDD, global developmental delay; ID, intellectual disability.

## 4 DISCUSSION

In the current study, through WES, one variant in the*MED23* gene was suggested for ID. This gene is a key regulator of the protein‐coding gene expression and is involved in adipogenesis, chromatin modification, neural differentiation, proliferation, smooth muscle cell differentiation, and tumorigenesis.^5^, ^22^, ^26^ Mutations in this gene have been associated with several disorder, including cancer as well as, cardiovascular and neurodevelopmental diseases.^10^, ^27^, ^28^ Loss of function of the *MED23* gene in the early developmental stages causes neurological disorders, whereas somatic mutations and variations in expression levels, play roles in the development of different kind of cancer later in life, including breast cancer, lung cancer, liver cancer, esophageal cancer, and Colorectal cancers.^22^, ^28^, ^30^

So far, only four variants in the*MED23* have been associated with an autosomal recessive form of ID and refractory epilepsy.^5^, ^9^, ^10^ The summary of *MED23* variants is summarized in Table 1. Hashimoto et al^5^ first associated *MED23* with a nonsyndromic autosomal recessive intellectual disability in five affected individuals of a consanguineous Algerian family by using homozygosity mapping and linkage analysis. Their patients had mild to moderate ID, with no pathological physical brain imaging or electroencephalogram evidence. They found that the p.R617Q variant did not affect *MED23* expression levels, protein stability, architecture, or composition of the whole Mediator complex, but specifically impaired the response of the *JUN* and the *FOS*, immediate‐early genes to serum mitogens.^5^ The same gene was mutated in two brothers in a nonconsanguineous family with novel compound heterozygous mutations, 3656A>G and 4006C>T, in *MED23*.^9^ The patients had severe ID, spasticity, congenital heart diseases, brain abnormalities, and atypical electroencephalography. After these two reports, one patient from consanguineous parents had been reported with refractory epilepsy, which was associated with homozygous *MED23* mutation. The patient had an infantile onset of global developmental delay, microcephaly, truncal hypotonia, and refractory epilepsy. The report of Lionel et al^10^ showed that the seizure in the patient could be treated with a ketogenic diet.

In the current study, the patient manifested clinical symptoms similar to previously described cases, including developmental delay, microcephaly, hypotonia, severe intellectual disability, and spasticity. In addition, this case had speech delay that was not reported in previously reported cases.

The sequencing results revealed one novel homozygous variant, c.670C>G, in the *MED23* gene. According to the ACMG guideline this variant was classified as a VUS, but based on several line of evidences suggested to be likely pathogenic, including clinical observation, segregation analysis, different amino acid properties, and location of the variant at the early exonic position.^10^

In conclusion, the result of this study showed the efficacy of WES in the detection of the candidate variants in ID.

## CONFLICT OF INTEREST

The authors declare that they have no financial or other conflicts of interest in relation to this research and its publication.

## AUTHOR CONTRIBUTION

FHG: involved in genetic testing and provided scientific support for writing and editing the manuscript. MF: involved in genetic testing and technical support. SMBT: involved in clinical evaluation and diagnosis. MM supervised the study and revised the manuscript for important intellectual content.

Accession Number: The variant obtained ClinVar accession number SCV000590917.1.
